# Genetic diversity and risk factors for the transmission of antimicrobial resistance across human, animals and environmental compartments in East Africa: a review

**DOI:** 10.1186/s13756-020-00786-7

**Published:** 2020-08-06

**Authors:** Bugwesa Z. Katale, Gerald Misinzo, Stephen E. Mshana, Harriet Chiyangi, Susana Campino, Taane G. Clark, Liam Good, Mark M. Rweyemamu, Mecky I. Matee

**Affiliations:** 1 Department of Microbiology and Immunology, School of Medicine, Muhimbili University of Health and Allied Sciences, Dar es Salaam, Tanzania; 2 Tanzania Commission for Science and Technology, Dar es Salaam, Tanzania; 3 SACIDS Foundation for One Health (SACIDS), Sokoine University of Agriculture, Morogoro, Tanzania; 4 Department of Veterinary Microbiology, Parasitology and Biotechnology, College of Veterinary Medicine and Biomedical Sciences, Sokoine University of Agriculture, Morogoro, Tanzania; 5 Department of Microbiology and Immunology, Catholic University of Health and Allied Sciences, Mwanza, Tanzania; 6 Faculty of Infectious and Tropical Diseases, London School of Hygiene and Tropical Medicine, Keppel Street, London, WC1E 7HT UK; 7 Faculty of Epidemiology and Population Health, London School of Hygiene and Tropical Medicine, Keppel Street, London, WC1E 7HT UK; 8 Department of Pathobiology and Population Sciences, Royal Veterinary College, London, UK

**Keywords:** Genetic diversity, Risk factors, Antimicrobial resistance, Human-animal-environment, East Africa

## Abstract

**Background:**

The emergence and spread of antimicrobial resistance (AMR) present a challenge to disease control in East Africa. Resistance to beta-lactams, which are by far the most used antibiotics worldwide and include the penicillins, cephalosporins, monobactams and carbapenems, is reducing options for effective control of both Gram-positive and Gram-negative bacteria. The World Health Organization, Food and Agricultural Organization and the World Organization for Animal Health have all advocated surveillance of AMR using an integrated One Health approach. Regional consortia also have strengthened collaboration to address the AMR problem through surveillance, training and research in a holistic and multisectoral approach. This review paper contains collective information on risk factors for transmission, clinical relevance and diversity of resistance genes relating to extended-spectrum beta-lactamase-producing (ESBL) and carbapenemase-producing Enterobacteriaceae, and Methicillin-resistant *Staphylococcus aureus (MRSA)* across the human, animal and environmental compartments in East Africa.

**Main body:**

The review of the AMR literature (years 2001 to 2019) was performed using search engines such as PubMed, Scopus, Science Direct, Google and Web of Science. The search terms included ‘antimicrobial resistance and human-animal-environment’, ‘antimicrobial resistance, risk factors, genetic diversity, and human-animal-environment’ combined with respective countries of East Africa. In general, the risk factors identified were associated with the transmission of AMR. The marked genetic diversity due to multiple sequence types among drug-resistant bacteria and their replicon plasmid types sourced from the animal, human and environment were reported. The main ESBL, MRSA and carbapenem related genes/plasmids were the ^*bla*^CTX-Ms (45.7%), SCCmec type III (27.3%) and IMP types (23.8%), respectively.

**Conclusion:**

The high diversity of the AMR genes suggests there may be multiple sources of resistance bacteria, or the possible exchange of strains or a flow of genes amongst different strains due to transfer by mobile genetic elements. Therefore, there should be harmonized One Health guidelines for the use of antibiotics, as well as regulations governing their importation and sale. Moreover, the trend of ESBLs, MRSA and carbapenem resistant (CAR) carriage rates is dynamic and are on rise over time period, posing a public health concern in East Africa. Collaborative surveillance of AMR in partnership with regional and external institutions using an integrated One Health approach is required for expert knowledge and technology transfer to facilitate information sharing for informed decision-making.

## Background

The emergence and the spread of antimicrobial resistance (AMR) presents a global challenge to diseases control. AMR can occur naturally following exposure to antimicrobial agents in the management of veterinary and human clinical cases [1]. The acquisition and spread of AMR can be attributed to ecological connectivity and presence in the community, of a previously acquired resistance gene, which can be denoted as a founder effect [2]. In resource-limited settings, the availability of antibiotics over the counter and without prescription mainly for self-treatment of suspected infections contributes to AMR. Of particular concern is the emergence of multi-drug resistant (MDR) bacteria such as methicillin-resistant *Staphylococcus aureus* (MRSA) and extended spectrum beta-lactamase (ESBL)-producers. For instance, MDR invasive non-typhoidal Salmonella (iNTS) disease poses a major challenge to the clinical management of infections in resource-limited settings especially as alternative more effective antibiotics remain either unaffordable or simply unavailable for majority of patients [3].

In East Africa, resistance to commonly used antibiotics have been reported in humans [4–11], livestock [12, 13], wildlife [14] and environment [15, 16]. There is a worrying trend of increasing prevalence of AMR [17]. In recent years, the numbers of infections due to the ESBL-producing Enterobacteriaceae (ESBL-PE), MRSA, and carbapenem resistance (CAR) have increased, and they are now been recognized as “infections of concern” to East African and wider international populations. The carbapenem-based antibiotics, which are considered as “antibiotics of last resort” or “last-line agents”, have been compromised by the emergence of MDR Gram-negative bacteria [18]. In Sub-Saharan African countries, infection, and control measures against MRSA amplify the challenges in dealing with the AMR epidemic [19]. MRSA is primarily mediated by the *mecA* gene, located in staphylococcal cassette chromosome mec (SCCmec) that codes for a 78 kDa penicillin-binding protein (PBP2a), that has decreased affinity to methicillin and all beta-lactam antibiotics [20, 21]. Likewise, ESBL-PE possess a threat [22], associated with pediatric septicemia and urinary tract infections [23]. The largest ESBL group are the mutants ^*bla*^TEM and ^*bla*^SHV β-lactamases followed by ^*bla*^CTX-M enzymes [24]; others include ^*bla*^OXA-type β-lactamases, PER, VEB, GES.BES.TLA, SFO, IBC groups [24]. The co-emergence of ESBLs and carbapenemase-encoding plasmids facilitate selection for other resistance determinants for other antimicrobial classes, including aminoglycosides and fluoroquinolones, a key feature that fosters the spread of MDR in Enterobacteriaceae [25]. Further, a plasmid with ESBL determinants can also carry aminoglycosides and ciprofloaxcin resistance (CR) determinants. Such co-existence of resistance mutations on plasmids or ESBL related genes is not uncommon [26].

In East Africa, CR has been reported in Enterobacteriaceae and other gram-negative bacteria [9, 17, 27–32]. The high prevalence of CAR Enterobacteriaceae among *Klebsiella pneumoniae* and *Escherichia coli* present a challenge against treatment of the carbapenemase-producing Enterobacteriaceae infections [33]. Despite the fact that colistin is effective in the treatment of Gram-negative infections, it is toxic with a poor treatment outcome [34]. Resistance due to carbapenem antibiotics is mediated through the production of carbapenemases *beta*-lactamases enzymes such as veron integron metallo-beta-lactamases, imipenemase, *K. pneumoniae* carbapenemases, oxacillinase-48, and New Delhi Metallo-beta-lactamase_1, which are encoded by “^*bla*^VIM”, “^*bla*^IMP”, “^*bla*^KPC”, “^*bla*^OXA-48”, and “^*bla*^NDM” genes respectively [9].

In resource-limited settings, knowledge of the burden, distribution, and diversity of resistance genes in humans, animals and the environment is scarce [35]. Despite increasing research on AMR in such settings, few studies have adopted an integrated “One Health” (OH) approach to understand the transmission dynamics of AMR across humans, animals and the environment. Most AMR studies in resource-limited settings are human-focused [36], and constitute the majority of current knowledge [35]. Nevertheless, major policy reforms have been implemented in developing countries to generate a collective response to address AMR and antimicrobial use, set within a holistic and multisectoral One Health framework. In Tanzania, the SACIDS Foundation for One Health has focused on the burden of AMR in the southern African region, and has been providing support in the local and global fight against AMR. The SACIDS Foundation for One Health in partnership with the American Society for Microbiology implemented the Tanzania National Action Plan on AMR through the Fleming Fund Initiative to support AMR surveillance strategy. This strategy attempts to address the major gaps in AMR data collection and analysis, whilst strengthening antimicrobial stewardship. Furthermore, the Africa Centers for Disease Control and Prevention Framework for AMR in collaboration with public health institutions and leaders from human and animal health sectors have established a network for the surveillance of AMR. This network aims to measure, prevent, and mitigate the effects arising from drug resistance organisms.

Our paper presents and analyzes information on AMR from published articles in East Africa in order to provide an overview of risk factors for AMR transmission and genetic diversity of ESBL, MRSA and CAR resistance genes, as well as a consideration of the role of humans, animals and the environment in emergence and spread of AMR. Further, the review paper addresses the clinical relevance of ESBL, MRSA and CAR bacteria and initiatives which have been taken by regional consortia. These initiatives, in collaboration with external institutions, are addressing the AMR problem through an integrated One Health approach, and providing evidence-based information for decision making in the management of AMR clinical cases.

## Materials and methods

The review of the literature was performed using search engines such as PubMed, Scopus, Science Direct, Google, Web of Science. Literature search was extended to involve repositories and sector ministries websites; however, no additional information was found. Search terms included ‘antimicrobial resistance and human-animal-environment, risk factors and predictors’, ‘antimicrobial resistance in human and animal populations’, prevalence and genotypic characterization of antimicrobial resistance’, methicillin-resistant *Staphylococcus aureus*’, ‘beta-lactamase genes and phenotypes’ Carbapenems resistance genes; ‘antimicrobial resistance genetic diversity and human-animal-environment’ combined with respective countries in East Africa including Burundi, Kenya, Rwanda, Tanzania and Uganda. The inclusion criteria included articles published between January 2001 and December 2018, which characterized AMR genes using genotypic methods in animals, humans and the environment. The exclusion criteria involved studies that did not utilize genotypic methods to identify resistance genes and sequence types of the AMR genes in the bacterial isolates.

Non-English language articles from French Speaking African countries such as Rwanda and Burundi were also excluded. The data were extracted using a template set of characteristics, including bacteria species, their origin (e.g. human, animal and environmental compartments), the proportion of the resistance genes, genotypic methods used for identification of AMR genes, sequence/clones/lineage types, plasmid replicon type and country of isolation. Further, descriptive statistics was performed to indicate trends of ESBLs, CARs and MRSA genes for the screened articles. The proportions of AMR genes from various pathogens in humans, animals and environment were correlated all-over the time-period for the screened studies using STATA version 14 (StataCorp LP, College Station, TX, USA), taking into consideration the time-period of recovered isolates. A *p*-value of < 0.05 considered statistically significant.

## Results

### The risk factors for emergence and transmission of AMR in animals, humans and the environment

The risk factors associated with the transmission of AMR genes varied within and between countries (Table 1), and can be broadly divided into five major types: (a) human practices, (b) demographic factors, (c) history of diseases and co-morbidities, (d) antibiotic use, and (e) hospitalization. We consider each in turn.

**Table 1:** Risk factors for the emergence and transmission of AMR in East Africa

Antimicrobial resistance study	Risk factors investigated	Significant risk factors	Hosts	Country	Reference
Nasal carriage of methicillin-resistant *Staphylococcus aureus* among healthy under-5 children	age, sex, education, visit the hospital, antibiotic use	–	human	Tanzania	[37]
Multiple ESBL-Producing *E. coli* Carrying Quinolone and Aminoglycoside Resistance Genes	sex, location, animal type, breed, antibiotic use	animal type, breed, antibiotic use	animals	Tanzania	[12]
Resistant *E. coli* in three culturally diverse ethnic groups	Increased number of water sources, adherence to antibiotic withdrawal periods, shared water resources, consumption of unboiled (raw) milk,	increased number of water sources, shared water, consumption of unboiled (raw) milk	Human, animals	Tanzania	[38]
Antibiotic Resistance in *E. coli*	Higher-income, antibiotics use	higher income	human	Tanzania	[39]
Nasal Carriage of Methicillin-Resistant *Staphylococcus aureus*	Duration in health care services, history of antibiotic use, history of chronic illness, duration in health care services, profession, age, location of health facilities, wards,	location of health facilities, duration in health care services	human	Tanzania	[40]
Extended-Spectrum-Beta-Lactamase-producing Enterobacteriaceae	Prior admission, prior medication, currently admitted in the surgical ward, patient inside the room, over 4 days of hospitalization, currently on the antibiotic, currently on Ciprofloxacin, currently on Ceftriaxone, HIV positive, wound infection	prior admission, currently on the antibiotic, wound infection,	human	Tanzania	[41]
Commonly used antimicrobial agents in bacterial pathogens isolated from urinary tract infections	Age, inpatient, hospitalization in the last 12 months, UTI in last 12 months, Urinary catheter, urinary catheter in last 12 months, use of other Antibiotics in the previous 6 months, Ciprofloxacin use in the previous 6 months, third-generation Cephalosporin use in the previous 6 months	Hospitalized (inpatient), third-generation Cephalosporin use in the previous 6 months, ciprofloxacin used in the previous 6 months	human	Rwanda	[42]
Antimicrobial resistance patterns of phenotype Extended Spectrum Beta-Lactamase producing bacterial isolates	Age, sex, department, sample type, ward/clinic, bacteria isolate, condition at discharge, the period of admission (days),	longer hospital stay, condition at discharge	human	Tanzania	[43]
Antimicrobial susceptibility profiles of *E. coli* and *K. pneumoniae*	Age, sex, health centre level, location of health sub-district, HSD, history of admission, history of medical procedures, surgery, antibiotic use, use of gentamicin, use of ciprofloxacin, use of septrin	age, level of health facility, location of health sub-district, district of residence, undergoing medical procedures, use of septrin	human	Uganda	[10]
Faecal carriage of ESBL-Producing Enterobacteriaceae	Sex, age, place of residence (district), parent level of education, children groups, hospitalized children, nutritional status, weight-for-age-Z-score, Length-for-age-Z-score, eight-for-length-Z-score, use of antibiotics, HIV	younger age, HIV infection and use of antibiotics	human	Tanzania	[44]
Predictors of blaCTX-M-15 in varieties of *E. coli* genotypes from humans	Age, number of children, sex, location, antibiotic use, admission history,	age, history of antibiotic use, history of admission in the past 1 year	human	Tanzania	[45]
Faecal carriage of CTX-M extended-spectrum beta-lactamase-producing Enterobacteriaceae	Source of income, source of food, local herbal use, street children type, primary education	local herbal use, street children type	human	Tanzania	[46]
Antimicrobial Resistance Profiles and Clonal Relatedness of *Pseudomonas aeruginosa* Strains	age, gender, residence, antimicrobial source, ease in accessing over the counter, prescription availability, dose completion	self-medication, non-completion of dosage	human	Kenya	[47]
Methicillin-resistant staphylococcus aureus (MRSA) colonization among Intensive Care Unit (ICU) patients and health care workers	Age, sex, education, occupation, smoking habit, history of sickness in past year, being sick for more 3 times, being diabetic, illicit drug use,	sex, history of sickness in past year, being sick for more 3 times, being diabetic, illicit drug use	human	Tanzania	[48]
Inappropriate usage of selected antimicrobials	Sex, age, breed, place/origin (rural, urban)	place/origin (rural/urban), age, breed	animals	Uganda	[49]
Extended-spectrum-beta lactamases producing Enterobacteriaceae	Age in days, sex, admission, body temperature, oxygen saturation, skin pustule, umbilical discharge, history of antibiotic-baby, maternal fever, maternal antibiotics, stool ESBL	positive ESBL-PE colonization of the mother, history of antibiotic use,	human	Tanzania	[50]

### Human practices

Transmission of AMR has been associated with practices involved in food production and animal husbandry [1]. Such practices include the use of antimicrobials in feeds as growth-promoters and to prevent infections [51–53] . For instance, Basulira et al. [49] reported a higher median concentration of antimicrobials such as *beta*-lactam antibiotics in adult carcass beef compared to young carcass beef samples. Such high levels of beta-lactam in feeds might be attributed by the indirect use of β-lactam antibiotics and tetracycline in feeds, drenches, drinking water and feed additives in the fattening systems [49]. In the Maasai community, consumption of unboiled (raw) milk was associated with increased odds of carriage of *E. coli* resistant to single and multiple antibiotics [38]. The relationship between milk and AMR was linked to consumption of antibiotic-resistant bacteria in contaminated milk [38]. Nevertheless, the Maasai and other communities are at a higher risk of diarrhea and other infections, from other sources. They tend to be greater consumers of certain types of antibiotics, leading to specific types of AMR [36]. In general, the spread of drug resistance is driven by multiple factors, which are linked to human life activity and travel, animals and the food trade, wild animals, migration, transportation, as well as water and wind [54].

### Demographic factors

Demographic analysis of AMR is important to reduce any potential adverse health impact in a timely fashion to limit the spread of the resistant pathogens. In a human context, various studies have found an increased prevalence of *beta*-lactamase genes with age [37, 44, 45, 55]. In the community settings in Mwanza City, Tanzania, increasing age was reported as a predictor for the carriage of ESBL-PE [45]. Similarly, Muvunyi et al. [42] found an association between increased age with the prevalence of ^*bla*^CTX-M Gram-negative bacteria. Likewise, an increased carriage of ^*bla*^CTX-M Gram-negative bacteria in persons over 65-year-old due to repeated hospitalization has been reported [55]. Further, it was hypothesized that due to reduced immunity in adults over 65, they are more prone to infections caused by AMR bacterial strains [55]. In Uganda, the carriage of MDR isolates was significantly associated with age, particularly the age group between 15 and 44 years [10]. However, contrary to that, Tellevik and colleagues reported age equal to or below 12 months to be associated with ESBL carriage [44]. Furthermore, Tellevik and others reported that infants compared to older children were more likely to be colonized with ESBL positive bacteria, with the possibly that resistant strains were being transmitted from mothers to their babies [44].

### History of co-morbidities

A history of malnourishment, diseases such as diabetes, HIV and other infections, were found to be associated with colonization of AMR bacteria [44, 48]. For instance, diabetic patients have reduced immunity, predisposing such patients to MRSA colonization [48]. Similarly, individuals with HIV are more prone to infections and hence are more likely to be hospitalized and consume antimicrobials [44]. Similarly, Cotton et al. [56] reported the association between HIV and ESBL carriage. The weak immunity in HIV patients increases the opportunity to acquire various infections [57]. Therefore, HIV positive pregnant women have been recommended to use anti-retroviral drugs to reduce mother-to-child transmission [44]. Despite the association between HIV and ESBL carriage, the HIV status has not been well documented as a risk factor for ESBL carriage [44]. Further, malnourished children with impaired immunity are more vulnerable to infections, and therefore more likely to be treated with antibiotics [44]. Godfrey and colleagues reported increased colonization of superficial skin infection with MRSA due to reduced skin immunity to fight against MRSA isolates [48].

### History of antibiotics use

The previous use of antibiotics has been associated with the development of AMR [10, 12, 42, 44, 45, 50, 55]. The history of antibiotics use is the main driver in the development of AMR and hence selection pressure specific for the type of antibiotic and the bacterial species [55, 58–60]. In Tanzania, the history of antibiotic use and positive ESBL-PE colonization of mothers was associated with neonatal ESBL colonization in a tertiary hospital [50]. Moreover, a history of antibiotic use increased the risk of developing antibiotic resistance, where patients in Tanzania who were on treatment to at least one antibiotic had an increased risk of ^*bla*^CTX-M Gram-negative bacteria compared to those who were not on any antibiotics [55]. In Rwanda, previous use of ciprofloxacin and other antibiotics in the previous 6 months was a risk factor contributing to CR [42]. Such findings also have been reported in other sub-Saharan African countries, particularly in Cameroon and Guinea-Bissau, where previous use of antibiotics was associated with ESBL carriage in hospital settings [61, 62]. However, this report is contrary to Moremi and colleagues who reported non-significant relationship between antibiotic use and ESBL-producing Enterobacteriaceae carriage among street children dwelling in Mwanza city [46]. Such a relationship was also reported by Fleece et al. [39], who found non-significant association between antibiotic use or episodes of diarrhea and antibiotic resistance in rural settings in Tanzania. In general, if the ecological and cultural conditions favour bacteria transmission, AMR bacteria could emerge irrespective of antimicrobial use practices in human health [38]. However, the heterogeneity between studies on the association between the previous use of antibiotics and AMR may be attributed to differences in study designs and the statistical methods used in data analysis.

### Hospitalization

Prolonged stay in hospital and condition at discharge were significantly associated with ESBL producers [43]. The risk for AMR is increased especially in patients who had continued exposure to antibiotics during hospitalization [43]. For instance, Moremi and her colleagues reported that the ESBL-producing Enterobacteriaceae carriage was significantly higher on discharge than admission [63]. In other studies, being male, a history of sickness in the past year, and > 3 illnesses within a short time, were associated with MRSA colonization among patients [48]. Further, the level of available healthcare and facilities predisposed patients to AMR [10]. For instance, patients admitted at referral hospital such as Muhimbili National Hospital had higher ESBL carriage upon admission than those admitted to a District Hospital in Dar es Salaam [44]. This finding is also supported by Seni et al. [64] in which the resistance to third-generation cephalosporins in *E. coli, Klebsiella* spp., and other Enterobacteriaceae was higher in strains from a tertiary hospital compared to lower healthcare facilities. Similarly, at Bugando Medical Centre tertiary hospital in Mwanza, Tanzania, the MDR was higher in isolates from children than those from the district and regional hospitals [65]. In Moshi region, Tanzania, patients who came to the referral hospital at Kilimanjaro Christian Medical Centre were carrying more resistant bacteria than hospitalized patients [55]. This difference increased the risks to health workers, particularly nurses who had frequent patient contact, especially when compared to doctors [40]. Nevertheless, the association between MRSA carriage among healthcare workers and facilities might be attributed to differences in levels of commitment to control measures of infection between tertiary and regional hospitals [40]. For example, Moremi et al. [66], reported similarity of genotypes in the intensive care units due to frequent movement of healthcare workers or instruments, implying the need to revise cleaning and disinfection protocols, as well as having the necessary precautions to avoid hand and clothing contamination during clinical practices.

### Genetic diversity, clinical relevance and regional surveillance of AMR in East Africa

#### Genetic diversity of AMR genes

**Fig. 1:**
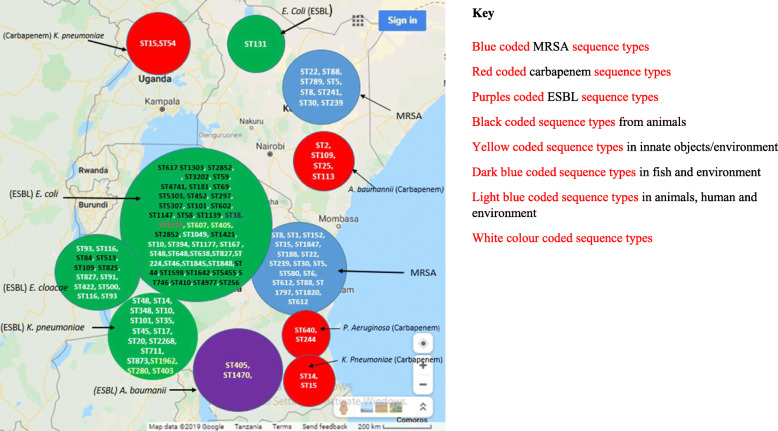
Geographical distribution of the sequence types from pathogens originated from various sources in the East Africa

Most studies in East Africa have reported considerable genetic diversity and differences in sequence types of AMR genes particularly for ESBL (Table 2, Fig. 1), MRSA (Table 3) [71–75], and CAR (Table 4). These are attributed to evolutionary events, including mutations, selection and gene transfer, in the AMR genes in MRSA, ESBL and carbapenem isolates. In East Africa, ESBL resistance genes have been isolated from *E. coli*, *Klebsiella* spp., *Proteus* spp., Enterobacter spp., *Pseudomonas aeruginosa*, Acinetobacter spp., *Klebsiella oxytoca*, *Proteus mirabilis*, *Enterobacter cloacae*, *Acinetobacter baumanii*, Salmonellae spp. and *E. cloacae* complex [6–8, 12, 15, 22, 23, 44, 50]. Further, proportions and range of antimicrobial resistance genes obtained from human, animals and environment differ (Table 5).

**Table 2:** The genetic diversity of extended–spectrum beta-lactamase (ESBL) genes

Pathogen	Source	Genotypic tools	Antimicrobial resistance genes	Sequence types/clones	Plasmid Replicon type	Phylogroups	Country	Reference	Time period for collection of isolates
*E. coli*	chickens	PCR	^*bla*^TEM (100%), ^*bla*^OXA-1 (75.0%), blaCMY-2 (62.5%), ^*bla*^CTX-M-8 (50%), ^*bla*^CTX-M − 9 (37.5%, ^*bla*^CTX-M − 1,15 (12.5%), ^*bla*^SHV (12.5%)		–	–	Tanzania	[67]	2016
*E. coli*	pigs, cattle, sheep, goats, dogs, chicken	WGS, MLST	*bla*OXA-1 & ^*bla*^TEM-1B (32%, 8/25), ^*bla*^OXA-1 (2/25, 8%), ^*bla*^TEM-1B 60% (15/25)	ST617, ST1303, ST2852, ST131,	IncFIA, IncFIB, IncFII, IncY,B/O/K/Z, IncX1, IncQ1, IncX3, IncX4, IncFIB(K), IncFIA	A, B1, B2, D	Tanzania	[12]	August and September 2014
*K. pneumoniae*	human	PCR, WGS, AFLP	^*bla*^TEM-63 (4/9;44.4%), ^*bla*^SHV-12 (0%), ^*bla*^SHV-2a (0%), ^*bla*^CTX-M-15 (5/9;55.6%),		–	–	Tanzania	[23]	August 2001 to August 2002,
*E. coli*	human	PCR, WGS, AFLP	^*bla*^TEM-63 (4/25, 16%), ^*bla*^SHV-2a (0/25, 0%), ^*bla*^SHV-12 (0/25, 0%), ^*bla*^CTX-M-15 (5/25, 20%)		–	–	Tanzania	[23]	August 2001 to August 2002,
*Salmonellae*	human	PCR, WGS, AFLP	^*bla*^TEM-63 (3/17, 17.6%), ^*bla*^SHV-2a (2/17, 11.8%), ^*bla*^SHV-12 (2/17, 11.8%), ^*bla*^CTX-M-15 (1/17, 5.9%)			–	Tanzania	[23]	August 2001 to August 2002,
*K. pneumoniae*	Human (blood, wounds, urine)	PCR, WGS, PFGE, MLST	^*bla*^CTX-M-15 (70/92; 76%), ^*bla*^TEM-1, ^*bla*^TEM-104 (18%), ^*bla*^SHV-11 (3.2%), ^*bla*^TEM-176 (2%)	ST48, ST14, ST348, ST10,	IncFII, IncND, IncFIA	–	Tanzania	[22]	Between April 2009 and March 2010
*E. coli*	cattle	ERIC-PCR, WGS	^*bla*^TEM-1B, ^*bla*^OXA-1, ^*bla*^TEM-1A	ST1139, ST617, ST3202, ST59, ST4741, ST181, ST69, ST5303, ST452, ST297, ST5307, ST101, ST602, ST1147, ST58	IncFIB (AP001918), ColRNAI, IncFIA, IncFII, IncFIC(FII),, InQ1, IncP, IncFII(pCoo), IncB/O/K/Z, Col156, IncFIB(pB171), IncFIA(HI1),, IncFII (pSE11),, IncX1, IncR, Incl1, Col(MG828),	A, B1, D	Tanzania	[13]	2014
*E. coil*	human	ERIC-PCR, WGS	^*bla*^TEM-1B, ^*bla*^OXA-1, ^*bla*^TEM-1A		IncFIB (AP001918), ColRNAI, IncFIA, IncFII, ColBS512, IncFII(pCoo), IncFII(29), IncFIB(K),lnO2,IncX4, Col(MP18),Col8282,	–	Tanzania	[13]	2014
*K. pneumonia*	environment	PCR	^*bla*^CTX-M-1		–	–	Tanzania	[15]	February 2014
*E. coli*	environment	PCR	^*bla*^CTX-M-1 (100%), ^*bla*^CTX-M-9(5.9), other than ^*bla*^CTM-1 & ^*bla*^CTM-9 (17.6%)		–	–	Tanzania	[15]	February 2014
*K. pneumoniae*	Human (blood)	WGS	^*bla*^CTX-M-15, ^*bla*^SHV-1, ^*bla*^TEM-1B, ^*bla*^SHV-11, blaSHV-33, ^*bla*^SCO-1, ^*bla*^OXA-1, ^*bla*^SHV-28, ^*bla*^SHV-83, ^*bla*^SHV-27,	ST101, ST348, ST35, ST45, ST14, ST17, ST20, ST2268, ST711, ST873	IncFIA, IncFIB, IncR, IncFII, IncHI1B, IncFR	–	Tanzania	[50]	Between July and December 2016
*E. cloacae*	human	WGS	^*bla*^CTX-M-15, ^*bla*^TEM-1B, ^*bla*^OXA-1, ^*bla*^ACT, ^*bla*^ACT-7	ST93, ST116,	IncHI2A, IncHI2,	–	Tanzania	[50]	Between July and December 2016
*A. baumanii*	human	WGS	^*bla*^ADC-25, ^*bla*^NDM-1, ^*bla*^OXA-69, ^*bla*^OXA-58, ^*bla*^CARB-8,	ST405, ST1470	–	–	Tanzania	[50]	Between July and December 2016
*E. coli*	human	RT PCR, WGS	^*bla*^CTX-M-15, ^*bla*^CTX-M-14, ^*bla*^CMY-2	–	–	–	Tanzania	[44]	From August 2010 to July 2011
*K. pneumonia*	human	RT PCR, WGS	^*bla*^CTX-M-15,	–	–	–	Tanzania	[44]	From August 2010 to July 2011
*K. oxytoca*	human	RT PCR, WGS	^*bla*^CTX-M-15,	–	–	–	Tanzania	[44]	From August 2010 to July 2011
*E. cloacae complex*	human	RT PCR, WGS	^*bla*^CTX-M-15,	–	–	–	Tanzania	[44]	From August 2010 to July 2011
*E. cloacae*	inanimate surfaces and objects	PCR, DNA sequencing	–	ST84, ST513, ST109, ST825, ST827	–	–		[66]	Between December 2014 and September 2015,
*Citrobacter spp*	human	RT PCR, WGS	^*bla*^CTX-M-15,	–	–	–	Tanzania	[6]	between 1992 and 2010
*E. coli*	human	PCR, WGS	^*bla*^TEM-1, ^*bla*^SHV-1, ^*bla*^CTX-M-14, ^*bla*^CTX-M-15, ^*bla*^CTX-M-9, ^*bla*^CTX-M, ^*bla*^CTX-M-3, ^*bla*^CTX-M-1, ^*bla*^SHV-5, ^*bla*^SHV-12, ^*bla*^TEM-52, ^*bla*^TEM-125, ^*bla*^TEM-50, ^*bla*^TEM-78, ^*bla*^TEM-109, TEM − 152, ^*bla*^TEM-158, ^*bla*^TEM-103, ^*bla*^CMY-2, ^*bla*^CMY-1, ^*bla*^OXA-1	–	–		Kenya	[68]	September and October 2009
*E. coli*	dogs, cats,	PCR, WGS, multiplex PCR	^*bla*^CTX-M-15 (dogs,47/216;22%), ^*bla*^CTX-M-15(cats, 2/50;4%), ^*bla*^OXA-1(dogs,47/216;22%), ^*bla*^OXA-1 (cats, 2/50;4%),	ST131	IncFIA, IncFIB, Incl1, IncFIA/FIB,	A, B1, B2	Kenya	[68]	September and October 2009
*E. coli*	human	PCR, multiplex PCR, WGS	^*bla*^CTX-M-154 (4/23;17%), ^*bla*^OXA-1(4/23;17%)	ST131	IncFIB	–	Kenya	[68]	September and October 2009
*E. coli*	human	PCR	^*bla*^CTX-M, ^*bla*^TEM	–	–	–	Kenya	[7]	March 2009 to February 2010
*K. pneumoniae*	human	PCR	^*bla*^CTX-M, ^*bla*^SHV, ^*bla*^TEM	–	–	–	Kenya	[7]	March 2009 to February 2010
*E. coli*	human	PCR, WGS	^bla^CTX-M-15, ^*bla*^CMY-2	–	–	–	Uganda	[41]	May 2010 to July 2011
*E. coli*	human	PCR	^*bla*^CTX-M, ^*bla*^SHV, ^*bla*^TEM	–	–	–	Uganda	[41]	May 2010 to July 2011
*E. coli*	human	PCR	^*bla*^CTX-M, ^*bla*^SHV, ^*bla*^TEM	–		–	Uganda	[41]	May 2010 to July 2011
*E. coli*	Fish	PCR	^*bla*^TEM (52%), ^*bla*^SHV (36%), ^*bla*^CTX 9.7%.		–		Tanzania	[47]	2017
*E. coli*	Fish	WGS	^*bla*^CTX-M-15 (9/11;81.8%), ^*bla*^OXA-1 (6/11;54.5%),	ST-38, ST-5173	IncI1, IncY,	E, B1	Tanzania	[69]	between July and September 2015
*E. coli*	Environment	WGS	^*bla*^CTX-M-15 (12/13;92.3%), ^*bla*^OXA-1 (1/13;7.7%)	ST38, ST-2852, ST-1049, ST-1421, ST-131, ST-10, ST-394, ST-1177, ST-58, ST-167, ST-48, ST-5173	IncY, IncI1, IncP, IncFII, IncFIA, IncFIB, IncQ1	B1, A, B2, E,	Tanzania	[69]	between July and September 2015
*E. coli*	inanimate surfaces and objects	PCR, DNA sequencing	^*bla*^CTX-M-15	ST607, ST405	–	–	Tanzania	[66]	December 2014 and September 2015,
*K. pneumoniae*	inanimate surfaces and objects	PCR, DNA sequencing	^*bla*^CTX-M-15	ST1962, ST280, ST403	–	–	Tanzania	[66]	December 2014 and September 2015,
*E. cloacae*	Fish	WGS	^*bla*^CTX-M-15, ^*bla*^TEM-1B, ^*bla*^ACT-15, ^*bla*^OXA-1, ^*bla*^MIR-3,	ST91, ST422, ST500	IncFII,IncFIB, IncFIB(K), IncFII, IncR	–	Tanzania	[69]	between July and September 2015
*K. pneumoniae*	Fish	WGS	^*bla*^CTX-M-15, ^*bla*^OXA-1, ^*bla*^SHV-11, ^*bla*^TEM-1B,	–	IncFII,IncFIB(K),IncHI1B,	–	Tanzania	[69]	between July and September 2015
*E. coli*	Animals	WGS		ST256, ST1303, ST1421, ST617, ST38, ST131, ST44, ST1598, ST1642, ST2852, ST5455, ST746, ST410, ST4977	IncFIA, IncFIB, IncFII, IncY,B/O/K/Z,IncX1, IncQ1, IncX3,IncIFIA, X4, IncFIB(K),	B1, A, D, B2,	Tanzania	[12]	between August/September 2014
*E. coli*	human	WGS	^*bla*^CTX-M-15, ^*bla*^OXA-1, ^*bla*^TEM-1B,	ST131, ST405, ST617, ST648	IncFIA, IncFIB, IncFII, IncI2, IncI1, Col156, IncQ1, IncY, IncQ, Col (BS512)	–	Tanzania	[50]	Between July and December 2016
*A. baumanii*	human	WGS	^*bla*^ADC-25, ^*bla*^OXA-69, ^*bla*^NDM-1, ^*bla*^OXA-58	ST405, ST1470,	–	–	Tanzania	[50]	Between July and December 2016
*E. cloacae*	human	WGS	blaCTX-M-15, ^*bla*^TEM-1B, ^*bla*^OXA-1, ^*bla*^ACT, ^*bla*^ACT-7	ST116, ST93	IncHI2A, IncHI2	–	Tanzania	[50]	Between July and December 2016
*K. pneumoniae*	human	WGS	^*bla*^CTX-M-15, ^*bla*^SHV-1, ^*bla*^TEM-1B, ^*bla*^SHV-11, blaSHV-33, ^*bla*^SCO-1, ^*bla*^OXA-1, ^*bla*^SHV-28, ^*bla*^SHV-83, ^*bla*^SHV-27,	ST101, ST348, ST35, ST45, ST48, ST14, ST17, ST20, ST2268, ST711, ST873	ncFIA, IncFIB, IncR, IncFII, IncHI1B,		Tanzania	[50]	Between July and December 2016
*E. coli*	human	PCR, DNA sequencing	^*bla*^CTX-M-15, ^*bla*^TEM-1	ST131, ST405, ST638, ST38, ST827, ST224, ST648, ST46, ST1845, ST1848	ncFIA, ncFIB, ncFII, ncFrepB, ncFIA- FIB	B2, D	Tanzania	[70]	2011
*K. pneumoniae*	human	WGS	^*bla*^SHV, ^*bla*^LEN, ^*bla*^OKP, ^*bla*^TEM, ^*bla*^CTX-M-15, ^*bla*^OXA, ^*bla*^SCO, ^*bla*^DHA1, ^*bla*^CARB, ^*bla*^NDM-1	ST15, ST54,	–	–	Kenya	[27]	1994–2002

*WGS* Whole genome sequencing

*AFLP Amplified Fragment Length Polymorphism

*PFGE Pulse field gel electrophoresis

*MLST Multi-Locus sequence typing

*ERIC-PCR Enterobacterial Intragenic Consensus-Polymerase Chain Reaction fingerprinting

**Table 3:** Genetic diversity of Methicillin Resistance *S. aureus* (MRSA) in the East Africa region

Pathogens	Source	Genotyping tools	Proportion of MRSA genes, n (%)	Phylogroups/sequence types	Lineages/spa type	Country	Reference	Time period for collection of isolates
MRSA	human	WGS, MLST	*mec*A (10/30;33.3%)	13 sequence types (ST-8, ST-1, ST-152, ST-15, ST-1847, ST-188, ST-22, ST-239, ST-30, ST-5, ST-580, ST-6		Tanzania	[71]	August 2013 to August 2015
MRSA	human	Multiplex PCR	SCC*mec* I (1/69;1.4%), SCC*mec* II (52/69; 75.4%), SCC*mec* IV (2/69; 2.9%)	**–**		Kenya	[72]	2005–2007
MRSA	human, environment	Multiplex PCR	SCC*mec* I (22/41;54%), SCC*mec* V (6/41;15%), SCC*mec* IV (3/41;7%)	**–**		Uganda	[73]	November, 2009 and February, 2010
MRSA	human	PCR, MLST	SCC*mec*A (24/24:100%)	ST88, ST 1797, ST1820	t064, t104, t1855, t186, t667, t690, t7237, t7231,	Tanzania	[74]	Between January and December 2008
MRSA	human	PCR, MLST	SCC*mec* type V (3/30;10%), SCC*mec* type IV (12/30;40%), SCC*mec* type III (13/30;43.3%), SCC*mec* type I (1/30;3.3%), SCC*mec* type II (1/30;3.3%)	ST22, ST88, ST789, ST5, ST8, ST241, ST30,	t037, t13149, t005, t022, t1339, t648, t345, t318, t293, t2029, t852, t689, t104, t1476, t13150, t091, t3202, t9622	Kenya	[75]	Between January 2010 and July 2013
MRSA	human	PCR, Multiplex PCR	SCC*mec* V (33.3%, 8/24), SCC*mec* IV (4.2% (1/24) SCC*mec* II (8.3% (2/24), SCC*mec* type 1 (16.7%, 4/24)	–	t645, t4353, t064, t355, t4609, t10277	Uganda	[76]	September 2011 to April 2012
MRSA	human	PCR, Multiplex PCR, WGS	SCC*mec* type I (56.4%), SCC*mec* type IV (17.9%)	–	–	Rwanda	[97]	
MRSA	human	WGS	SCC*mec* III (6/6; 100%)	ST239	t037	Kenya	[77]	Between the 11th July and 7th November 2011,
MRSA	cattle	PCR, Multiplex PCR, PFGE	SCC*mec* type V (21/23; 91.3%)	–	t7753, t1398, t2112, t3992, t127	Uganda	[78]	July to August 2013
MRSA	human	PCR, WGS	SCC*mec*A	ST612	t690	Tanzania	[66]	Between December 2014 and September 2015
MRSA	human	PCR, WGS	SCC*mec* type IV (40.5%, 17/42), SCC*mec* type I (38.1%, 16/42), SCC*mec* types I, II and III (50%,21/42)	–	t064 (19%, 8/42), t037 (12%, 5/42). t002, t037, t064, t4353 and t12939	Uganda	[16]	Between February and October 2011

**Table 4:** Genetic diversity of Carbapenem-resistant genes in the East Africa region

Pathogen	Source	Genotyping tools	Proportion of carbapenemase resistance genes	Phylogroups/sequence types	Lineages/cluster	Country	Reference	Time period for collection of isolates
*P. aeruginosa*	human, environment	PCR, WGS	blaIMP (36%, 9/25), blaVIM1 (32%, 8/25), blaSPM (16%, 4/25), blaNDM1 (4%, 1/25)	–	–	Uganda	[31]	Between February 2007 and September 2009
*A. baumannii*	human, environment	PCR, WGS	blaOXA-24 (7%, 1/15), blaVIM-1 (13%, 2/15), blaOXA-58 (13%, 2/15), blaOXA-23 (60%, 9/15)	–	–	Uganda	[31]	Between February 2007 and September 2009
*P. aeruginosa*	human	PCR, WGS, PFGE	blaVIM-2 (13.7%; 57/416), blaVIM-1 (13.7%; 57/416)	–	MBLA, MBLAR, MBLB,	Kenya	[32]	2006 and 2007
*P. aeruginosa*	human	PCR, WGS, PFGE	^bla^IMP (12/49;24.5%), ^bla^VIM (9/28; 32.1%), blaOXA_48 (2/11;18.2%), blaKPC (1/8; 12.5%), blaNDM (1/8; 12.5%)	–	–	Tanzania	[9]	Between 2007 and 2012
*P. aeruginosa*	human	PCR, WGS	blaVIM-2	ST640, ST244		Tanzania	[41]	May 2010 to July 2011
*K. pneumoniae*	human	PCR, WGS, PFGE	^bla^IMP types (49/227; 21.6%), ^bla^VIM types (28/227; 12.3%), ^bla^OXA_48 11/227 (4.9%), ^blaKPC^ (8/227; 3.5%), ^blaNDM^ (7/227;3.1%), ^bla^NDM-1, ^bla^IMP, ^bla^NDM-1,	ST14, ST15	–	Tanzania,	[9]	Between 2007 and 2012
*K. pneumoniae*	human	PCR, WGS, PFGE	^bla^NDM-1 (4/35; 11.4%), ^bla^VIM (16/35;45.7%), ^bla^IMP (5/35;8.16%), ^bla^KPC (3/35;8.6%), ^bla^OXA-48 (7/35;20%),	–	–	Uganda	[17]	between January, 2013 and March, 2014
*K. pneumoniae*			^bla^VIM (17/43;39.5%), ^bla^OXA-48 (5/13;38.5%)	–		Uganda	[30]	January, 2013 and March, 2014
*E. coli*	human	PCR	^bla^IMP (19/32;59.4%), ^bla^NDM (0/32;0%), ^bla^VIM (4/32;12.5%), ^blaOXA^_48 (3/32;9.4%), ^blaKPC^ (4/32; 12.5%), blaNDM (2/32;6.3%)	–	–	Tanzania,	[9]	Between 2007 and 2012
*E. coli*	human	PCR	^bla^NDM-1 (0/19; 0%), ^bla^VIM (1/19;5.3%), ^bla^IMP (6/19;31.6%), ^bla^KPC (4/19;21.1%), ^bla^OXA-48 (8/19;42.1%)	–	–	Uganda	[17]	between January, 2013 and March, 2014
*E. coli*	human	PCR	^bla^VIM (20/43;46.5%), ^bla^OXA-48 (6/13;46.2%)	–	–	Uganda	[30]	January, 2013 and March, 2014
*A. baumannii*	human	PCR, PFGE, WGS	^bla^IMP types (3/3;100%),^bla^VIM (0/3; 0%)^bla^OXA_48 (0/3; 0%), ^bla^KPC (0/3;0%), ^bla^NDM (0/3;0%)	–	–	Tanzania,	[9]	Between 2007 and 2012
*A. baumannii*	human	PCR, PFGE, MLST	ISAba1-blaOXA-23, blaOXA-51-like, ^*bla*^ADC, blaNDM-1, ADC-57.	ST2, ST109, ST25, ST113	European clone II (ECII)	Kenya	[28]	January 2009 to August 2010
*Salmonella spp*	human	PCR	^bla^IMP types (1/2;50%),^bla^VIM(1/50; 50%)	–	–	Tanzania	[9]	Between 2007 and 2012
*K. oxytoca*	human	PCR	^bla^IMP types (3/5;60%),^bla^OXA481/5;20%),^bla^NDM1/5;20%)	–	–	Tanzania,	[9],	Between 2007 and 2012
*K. oxytoca*	human	PCR	^bla^NDM-1 (1/1; 100%), blaVIM (0/1;0%), ^bla^IMP (0/1;0%),^bla^KPC (0/1;0%), ^bla^OXA-48 (1/1;100%)	–	–	Uganda	[17]	between January, 2013 and March, 2014
*C. freundii*	human	PCR	^bla^IMP types (2/4;50%), ^bla^VIM (1/4;25%), ^bla^OXA_48 (1/4;25%),	–	–	Tanzania	[9]	Between 2007 and 2012
*C. freundii*	human	PCR	^bla^NDM-1 (1/1; 12.5%), ^bla^VIM (0/1;0%), ^bla^IMP (0/8;0%), ^bla^KPC (0/1;0%), ^bla^OXA-48 (0/1;0%),	–	–	Uganda	[17]	between January, 2013 and March, 2014
*P. aeruginosa*	human	PCR	^bla^IMP types (12/25;48%), ^bla^VIM (9/25;36%), ^bla^OXA_48 (2/25;8%),^bla^KPC (1/25;4%),^bla^NDM (1/25; 4%)	–	–	Tanzania	[9]	Between 2007 and 2012
*Enterobacter* spp.	human	PCR	^bla^NDM-1 (1/5; 20%), ^bla^VIM (1/5;20%), ^bla^IMP (0/1;0%), ^bla^KPC (1/5;0%), ^bla^OXA-48 (2/5;40%)	–	–	Uganda	[17]	between January, 2013 and March, 2014
*Pantoea agglomerans*	human	PCR	^bla^NDM-1 (0/1; 0%), ^bla^VIM (0/1;0%), ^bla^IMP (1/1;100%), ^bla^KPC (0/1;0%), ^bla^OXA-48 (0/1;0%)	–	–	Uganda	[17]	between January, 2013 and March, 2014
*Proteus mirabilis*	human	PCR	^bla^NDM-1 (0/2; 0%), ^bla^VIM (0/2;0%), ^bla^IMP (0/2;0%), ^bla^KPC (1/2;50%), ^bla^OXA-48 (1/2;100%)	–	–	Uganda	[17]	between January, 2013 and March, 2014
*Proteus mirabilis*	human	Multiplex PCR	^bla^VIM1/43;2.3%), ^bla^OXA-48 (1/13;7.7),	–	–	Uganda	[30]	September 2013 to June 2014,
*Serratia marcescens*	human	PCR	^bla^NDM-1 (0/3; 0%), ^bla^VIM (2/3;66.7%), ^bla^IMP (0/3;0%), ^bla^KPC (1/3;33.3%), ^bla^OXA-48 (0/3;0%)	–	–	Uganda	[17]	between January, 2013 and March, 2014
*Morganella morganii*	human	Multiplex PCR	^bla^VIM1/43; %2.3), ^bla^OXA-48 (0/13;0%),	–	–	Uganda	[30]	September 2013 to June 2014,
*Enterobacter sakazaki*	human	Multiplex PCR	^bla^VIM 0/13; 0%),^bla^OXA-48 (1/13; %7.7),	–	–	Uganda	[30]	September 2013 to June 2014,
*Stenotrophomonas spp*	human	Multiplex PCR	^bla^VIM (1/43;2.3%),^bla^OXA-48 (0/23;0%)	–	–	Uganda	[30]	September 2013 to June 2014,

**Table 5:** Proportions and range of antimicrobial resistance genes obtained from various pathogens in humans, animals and environment

Type of antimicrobial resistance genes	Range of ESBL genes in different studies (%)	Mean (n/N;%)
**Methicillin resistance*S. aureus*genes**		
SSCmec I	0–56.4	21.24
SSCmec II	0–8.3	1.45
SSCmec III	0–100	27.34
SSCmec IV	0–40.5	14.06
SSCmec V	0–91.3	18.70
SSCmec I,II,III	0–50	6.25
SSCmecA	0–100	16.66
**Extended spectrum beta-lactamase genes**		
TEM	0–100	26.7
OXA	0–75	15
CMY	0–62.5	4.81
CTX-M	0–100	45.68
SHV	0–36	7.72
OXA&TEM	0–32	2.46
**Carbapenames genes**		
IMP	0–100	23.75
VIM	0–66.7	18.84
SPM	0–16	0.64
NDMI	0–100	7.59
OXA	0–100	17.91
KPC	0–33.30	5.82

The genetic diversity of ESBL genes including ^*bla*^TEM, ^*bla*^OXA-1, ^*bla*^CMY-2, ^*bla*^CTX, ^*bla*^SHV, ^*bla*^SCO, ^*bla*^ACT, ^*bla*^ADC, ^*bla*^CARB, and ^*bla*^NDM have been collected from bacterial species sourced from animals, humans and the environment (Table 2). Such diversity of ESBL producing bacteria represents a huge challenge for local hospital infection control teams [22]. The ESBL, particularly ^*bla*^TEM-, ^*bla*^SHV-, and ^*bla*^CTX M-enzyme, exhibit a high degree of diversity with high levels of MDR [31, 44, 79, 80]. In East Africa, the ^*bla*^CTX-Ms have been reported to be the dominant enzymes among the ESBL genes [6–8, 15, 44, 69, 81]. However, this finding is contrary to reports by Armah [67] in Morogoro region, Tanzania in which the ^*bla*^TEM enzymes followed by ^*bla*^OXA-and ^*bla*^CMY-2 were isolated in higher proportion in poultry. The ESBL are encoded by plasmids and mobile genetic elements and transferred to other bacteria [82]. The genetic diversity of plasmid replicons types in humans, animals and environmental compartments has been reported in *E. coli, K. pneumoniae* and *E. cloacae* ESBL carrying isolates (Table 2). The IncF plasmids have been found in *E. coli* producing ESBL genes collected from animals, humans and the environment [12, 13, 68, 69]. Further, the IncF plasmid types such as IncFIA, IncFIB, IncFII, IncFIB (K) have been reported in *E. coli* producing ESBLs in domestic and companion animals [12], humans [68], thereby indicating commonality of IncF plasmids types circulating in humans and animals. Such associations have also been reported in *K. pneumoniae* and *E. cloacae* ESBL carrying isolates originating from humans [22, 50, 69] and fish [69]. The IncF types such as IncFIB (AP001918), IncFIA, IncFII, IncFII (pCoo c) were shared between animals and humans [13], indicating the possibility of cross-species transmission of plasmid replicon type among ESBL-PE genes carrying isolates.

In East Africa, the distribution and the predominance of the MRSA genes (Table 3) varies between countries. In Uganda, the SCCmec type 1 and SCCmec type V were the most common and accounted for between “33.3 - 91.3%” prevalence [73, 76, 78]. In Kenya, the SCCmec type II MRSA and a pvl strain of MRSA were significant pathogens in patients with soft tissue infections presenting to hospitals [72]. Further, a cross sectional study in Kenya, reported predominance of SCCmec type V followed by SCCmecII genes in healthy students residing within the university residence halls [83]. Several sequence types (STs) of MRSA have been reported in East Africa [71, 74, 75, 77]. For instance, the ST88 and ST1797 have been reported to be the predominant sequence types causing wound infection and abscesses in clinical isolates in a tertiary hospital in Tanzania [74]. In Kenya, ST241 was the predominant clonal complex in various healthcare institutions in Nairobi [75]. Further, ST239 isolates were associated with hospital-acquisition in Kenya [77]. The MRSA carriage isolates have been confined to patients with burns due to a prolonged stay in the hospital [77]. MRSA isolates were recovered from wound swabs, pus, and nasal swabs in Lake Victoria, Tanzania, and found the spa types t690 and t7231, ST88 and ST1797 were dominant among clinical cases [74]. The two MRSA isolates from wounds were all typed as ST612 [11]. In Kenya, all the MRSA isolates collected from inpatients in the medical, surgical and gynecological wards of Thika Hospital were of spa type t037, linked to internal sources within the hospital [77].

A wide diversity of CAR genes including ^*bla*^IMP^*, bla*^VIM-1 ^*bla*^SPM-l, ^bla^NDM-1, ^*bla*^OXA-23 ^*bla*^OXA-24, ^*bla*^OXA-58, ^*bla*^KPC and novel ADC, ^*bl*a^ADC-57 have been identified in East Africa [28, 33] (Table 4), mostly isolated from clinical samples in hospital settings. However, there is no information available on occurrence of CP bacteria in livestock and their environment in the East Africa [33]. The genetic determinants of CP have been reported in *P. aeruginosa*, *A. baumannii*, *K. pneumoniae*, Salmonella spp., *K. oxytoca*, Enterobacter spp., *Pantoea agglomerans*, *P. mirabilis*, *Serratia marcescens*, *Pantoea agglomerans*, *Morganella morganii*, *Enterobacter sakazaki* and *Stenotrophomonas* spp. (Table 4). The prevalence of carbapenemase genes isolated from different bacterial isolates varies. For instance, Mushi et al. [9] reported variation in the prevalence of CP genes, in which the overall frequency of drug resistance loci in *E. coli* was 14% (32 isolates) followed by *K. pneumoniae* 10.57% (24 isolates), *P. aeruginosa* 10.13%, *K. oxytoca* 1.76%, *A. baumannii* 1.3%, *Citrobacter freundii* 0.88%, *S. marcescens* 0.88%, and *Salmonella* spp. 0.44%). In South Western Uganda only ^*bla*^VIM and ^*bla*^OXA-48 genes were detected among the carbapenemase-producing Enterobacteriaceae of clinical origin [30]. However, most studies have reported low prevalence of the ^*bla*^NDM-types. For instance, the ^*bla*^NDM-1 gene was found to be uncommon (2.6%) among the bacterial isolates [17], signifying the low prevalence of the New Delhi Metallo-β-lactamase 1 (NDM-1) types in the East Africa. The ^*bla*^NDM-1 genes have been reported in Kenya [28], Uganda [17, 31] and Tanzania [9, 31]. The ^*bla*^NDM-1 genes were for the first time isolated in India and then reported in Europe and have been identified in extensively drug-resistant *A. baumannii* [28], raising a concern in the transmission dynamics of these genes in East Africa. Poirel and colleagues reported identical PGFE patterns of *K. pneumoniae* similar to strain 05–506 identified in Sweden. Strain 05–506 was associated with intercontinental transmission of the ^*bla*^NDM-1, by the Indian population in Kenya, and air traffic between Europe and East Africa [80]. The ^*bla*^NDM-1 gene has been found co-existing with other CP genes, thereby explaining why these isolates are MDR [17]. In Kenya, the ^*bla*^NDM-1 carbapenem-resistant NDM-1-positive *K. pneumoniae* isolates were clonally related and expressed other ß-lactamases genes including the ^*bla*^CTX-M-15, ^*bla*^OXA-1, ^*bla*^OXA-9, ^*bla*^CMY-6 and aminoglycoside resistance methylase RmtC genes [80]. Further, all isolates that carried the ^*bla*^NDM-1 carbapenemase gene were clonally related and expressed many other resistance determinants, including β-lactamases ^*bla*^CTX-M-15, ^*bla*^OXA-1, ^*bla*^OXA-9, ^*bla*^CMY-6, and aminoglycoside resistance methylase RmtC [80]. In Tanzania, Mushi and colleagues found solitary and heterogenous MDR gram-negative bacteria having at least two carbapenemase genes [9].

### Trends in the carriage of antimicrobial resistance genes of the recovered isolates overtime period

**Fig. 2:**
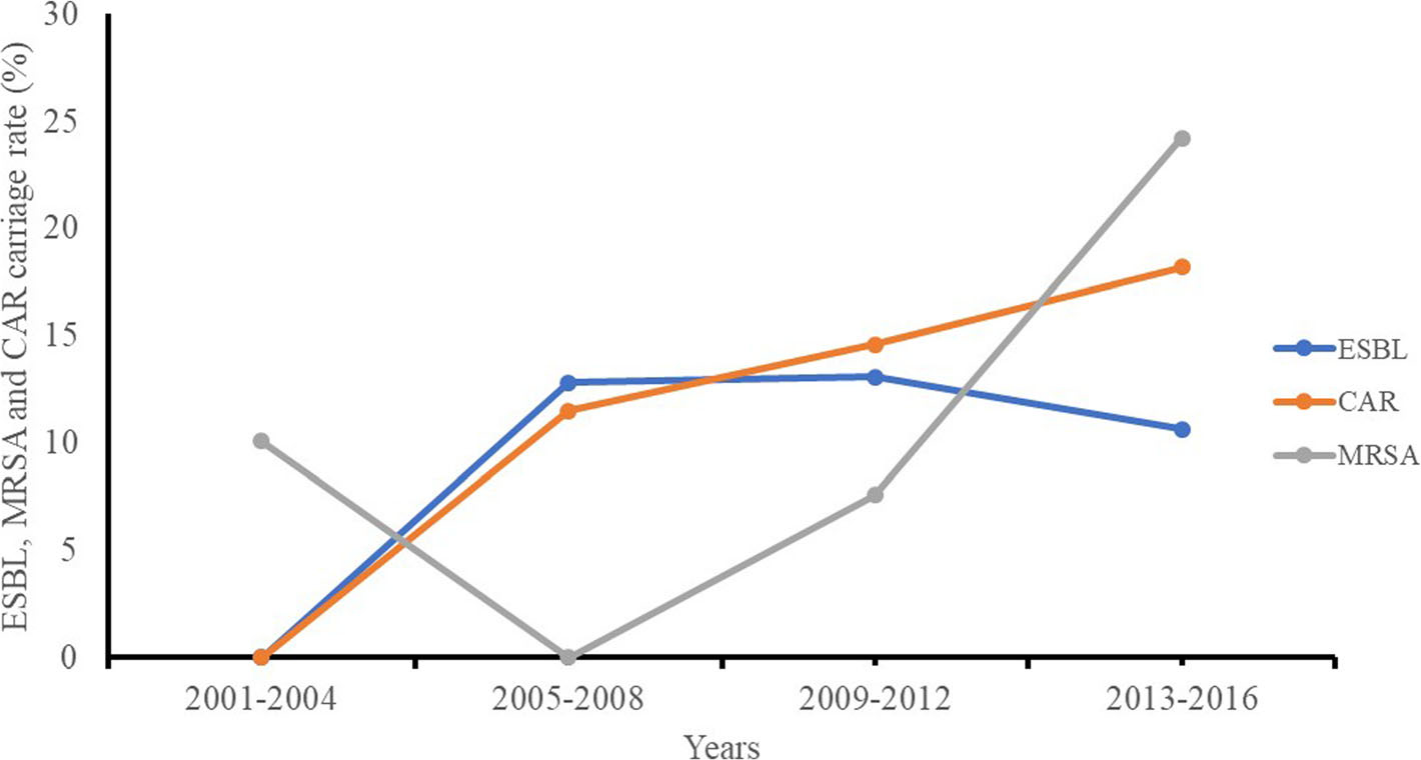
Trends in Extended Spectrum beta-lactamase, Methicillin resistance *Staphylococcus aureus* and carbapenem genes in recovered isolates in East Africa

Figure 2 represents the ESBL, MRSA, and CAR carriage rates over time, as established by a linear regression model using the values reported in screened studies conducted from 2001 to 2018 in East Africa. Over this period, the differences in ESBLs, MRSA, and carbapenem carriage rate in screened studies in East Africa were insignificant (*p-*value = > 0.05). The data obtained with period on carriage rate between ESBL and CAR [*p* value = 0.1; 95% CI = − 0.5 to 1.9%], ESBL and MRSA (*p* value = 0.9; 95% CI = 1.9 to 1.8%] and CAR and MRSA; [*p* value = 0.6; 95% CI = − 1.9 to 2.5%] were insignificant. However, AMR gene carriage in recovered isolates is on the rise and increased from 2001 to 2016, 2005 to 2016, and 2001 to 2012 for CAR, MRSA, and ESBLs respectively**.** The highest AMR genes carriage rates for CAR (18.2%), ESBL (13.1%), and MRSA (24.25%) were reported in studies conducted from 2013 to 2016, 2009–2012, and 2013–2016 respectively. The rise of ESBL, CAR, and MRSA carriage rates in East Africa pose a threat to public health particularly in the geographical areas where the rates are very high. A limitation of this analysis is the fact that these data were collected from unlinked cross-sectional studies conducted in different geographical areas in East Africa; consequently, some important information may have been missed.

#### Clinical relevance and outcome of the AMR genes in East Africa

The genetic diversity at antibiotic resistance loci could determine a population’s capacity to cope with future challenges of the antimicrobial drugs used for infection control [84]. The genetic diversities of AMR genes particularly ESBLs, MRSA, and CAR among clinical isolates in East Africa pose a public health threat. In East Africa, the ESBL, particularly TEM-, SHV-, CTX M-enzyme, exhibit a high degree of diversity [79, 80], associated with high levels of MDR [44]. The ESBLs are encoded by plasmids which also carries genes encoding for other drugs, limiting the antibiotic options in the treatment of ESBL producing organisms [85]. Carbapenems have been considered as the drug of choice for infection associated with ESBL-producing organisms. However, challenges remain as resistance to carbapenems has recently been reported, and there have been treatment failures [85]. The ESBL-producing organisms may appear susceptible to some extended-spectrum cephalosporins [85]. However, alternative treatment options to cephalosporins are limited in local settings. In Uganda, a high prevalence of carbapenem and CAR encoding genes among third-generation cephalosporins resistant Enterobacteriaceae have been reported in clinical isolates obtained from patients referred to Mulago hospital. Furthermore, high resistance rates in third-generation cephalosporin cefotaxime among urinary tract infection isolates have been recovered from pregnant women at Muhimbili National Hospital in Tanzania [86].

The high MRSA carriage rates among patients and health workers in the East Africa is a concern given poor infection prevention and control measures in our settings [87]. In Kenya, the trend in antibiotic susceptibility to MRSA declined between years 2014 to 2016, just like the methicillin susceptible counterparts [87]. In Kenya, MRSA is susceptible to linezolid, tigecycline, teicoplanin and vancomycin [87], quinupristin/dalfopristin, nitrofurantoin, and ampicillin/sulbactam [88]. Further, high resistance to commonly used antibiotics such as gentamycin, erythromycin, levofloxacin and tetracycline has also been reported in Kenya [88]. In Tanzania, high resistance rates among MRSA isolates to kanamycin, gentamicin, ciprofloxacin and trimethoprim-sulphamethoxazole have been reported [40]. Low resistance rates against MRSA isolates have been reported to mupiroaazcin and none toward linezolid, indicating that these two drugs can be used as a treatment options for MRSA infections in our settings [40]. In Uganda, the coexistence of hospital and community-associated MRSA have been fueled by pre-exposure to ampicillin and co-trimoxazole/or health care facilities in children [16]. Such coexistence presents a challenge for management of MRSA infection in outpatients.

#### Strategies and future for regional surveillance of AMR in the East Africa

The World Health Organization, the Food and Agriculture Organization of the United Nations and the World Organization for Animal Health has advocated for a holistic and multisectoral approach to address the AMR problem. This has been strengthened by the collaboration of regional consortia involving local and regional academic and research institutions in Africa, and a joint dedication of efforts to fight against infectious diseases in animals, humans and their environments. Regional consortia such as the SACIDS Foundation for One Health in collaboration with the London School of Hygiene and Tropical Medicine, London International Development Centre, Royal Veterinary College, Chatham House of the Royal Institute of International Affairs in the United Kingdom and the American Society of Microbiology of the United States, have partnered to address AMR problem through research and training. Strategically, surveillance is being supported by phenotypic and genomic data, to understand the flow of resistomes across human, animal and environmental compartments. For instance, research programs focused on genomic epidemiology of ESBLs producing *E. coli* in humans, animals and their environments are conducted by postgraduate students of the SACIDS Foundation for One Health at the Sokoine University of Agriculture and Muhimbili University of Health and Allied Sciences in Tanzania. This work is attempting to understand the transmission dynamics of AMR genes and their virulence [89], and will provide a model for the surveillance of AMR in Africa, including an exemplar of implementing global recommendations and local solutions. More generally, surveillance will be performed in line with the national AMR action plans to establish a nation-wide surveillance system for AMR, establish and build capacity for a national reference laboratory and designated laboratories for AMR surveillance using an integrated One Health approach. SACIDS Foundation for OH in collaboration with the American Society for Microbiology through the Fleming Fund initiative will implement the Tanzania National Action Plan for AMR launched in 2017. Further, through funding from the Medical Research Council in the United Kingdom, policy gaps, behaviour, socio-cultural and economic determinants for AMR in communities will be identified to provide evidence-based policy by policy makers to solve the AMR problem in Tanzania.

The implementation of effective and sustainable AMR surveillance programmes in Africa is hampered by a lack of infrastructure and other resources required to perform optimal surveillance [90]. Most laboratory facilities in resource-limited countries produce quality routine culture and susceptibility testing, which is both cost-effective and provides high-quality surveillance data. However, developing countries should consider incorporation of the whole genome sequencing in the diagnosis framework of drug resistance isolates. The MDR isolates present a challenge for management of clinical cases in resource-limited countries. Resistance to colistin, which is a last resort treatment for life-threatening infections caused by Enterobacteriaceae, including MDR pathogens has been reported in several countries and regions. Other infections such as MDR tuberculosis (MDR-TB) pose a threat to public health in the Sub Saharan African countries adding to the current long-standing pandemics of HIV in the region. Studies have found high diversity of mutations among MDR-TB patients, suggesting established and ongoing transmission of MDR-TB strains or multiple sources of infections [91]. However, it is unclear whether the role played by drug resistance genetic mutations in combination or alone could predict the disease manifestations or progression. Therefore, clinical studies have been proposed to evaluate the incorporation of whole genome sequencing in the diagnostic framework of drug resistance tuberculosis in these countries. It has been thought that the incorporation of whole genome sequencing as a diagnosis tool for MDR-TB in resource limited settings will improve the diagnosis and treatment of TB while providing stringent strain discrimination. Such incorporation would improve the detection of drug resistance mutations in TB patients, and to inform clinical and treatment decision making [92]. The information obtained from such clinical studies could support policy change in the adoption of molecular TB diagnostic algorithms in resource-limited countries.

## Conclusions

The high genotypic diversity of the AMR genes among the bacterial isolates might suggest possible exchange of strains or a flow of genes among different strains due to transfer by mobile genetic elements or multiple sources of resistance bacteria. Such levels of diversity prompt the calling for immediate interventions, including guidelines concerning antibiotics use and regulations governing their importation and sale. However, antibiotic use and regulation is likely to be a very complex system because other factors such as cultural and ecological conditions favour transmission of bacteria. There is a high likelihood that people will have AMR bacteria irrespective of how antibiotics are used. Therefore, control strategies against AMR need to be tailored, beyond antibiotic use and availability, to local practices that affect bacterial transmission. Moreover, the trend of ESBL, MRSA and CAR carriage rates is dynamic and are on rise over time period, posing a public health concern in East Africa. In addition to that, phenotypic and genotypic drivers of AMR should be investigated within an integrated One Health framework. Such investigations will inform transmission dynamics of resistomes across compartments, and facilitate information sharing for informed decision making; ultimately, reducing the spread of AMR genes in bacteria. Further, the application of advanced techniques such as whole genome sequencing for detection of AMR could cut a bridge between clinical research and care, so that case management and treatment decisions can be informed and personalised.

## Data Availability

Data and materials were available from peer reviewed articles published between January 2001 and December 2018 in East Africa.
